# Preclinical pharmacological effects of epimedium-derived interventions in osteoarthritis: a systematic review and meta-analysis

**DOI:** 10.3389/fphar.2026.1828529

**Published:** 2026-08-10

**Authors:** Xiang Ji, Rui Tang, Dongping Wan, Haodong Wu, Tingting Tang, Huanli Bao, Shuxin Yao, Chao Xu, Jianbing Ma

**Affiliations:** 1 Department of Knee Joint Surgery, Honghui Hospital, Xi’an Jiaotong University, Xi’an, China; 2 Xi’an Medical University, Xi’an, China; 3 Postgraduate Education, Guangxi University of Chinese Medicine, Nanning, China; 4 Officers College of the Chinese People’s Armed Police Force, Chengdu, Sichuan, China

**Keywords:** animal experiment, botanical drug, epimedium, inflammation, osteoarthritis

## Abstract

**Background:**

The genus *Epimedium* (Berberidaceae) is widely used in traditional medicine. Recent studies indicate that its botanical metabolites (such as icariin) exhibit anti-inflammatory and chondroprotective potential in osteoarthritis (OA) models. However, a comprehensive analysis remains lacking.

**Methods:**

This study was registered in PROSPERO (CRD420251143826). Chinese and English databases (PubMed, Web of Science, Embase, FMRS, VIP, CNKI, WanFang) were searched to collect preclinical controlled trials evaluating *Epimedium* extracts or their botanical metabolites for the treatment of OA in Murine subjects. The risk of bias was assessed using the SYRCLE tool, and data were synthesized using RevMan 5.4 and Stata 19.0.

**Results:**

Thirteen studies comprising 238 Murine subjects were included. Interventions utilizing *Epimedium*-derived botanical metabolites significantly improved Pelletier scores and thermal paw withdrawal latency (PWL). Concurrently, they decreased the levels of serum IL-6 and MMP-13, as well as intra-articular IL-1β, IL-18, and MMP-1. Favorable pharmacological trends were also observed for serum TNF-α, intra-articular MMP-13, and the mechanical paw withdrawal threshold (PWT). Subgroup analysis identified the route of administration as a significant source of heterogeneity for intra-articular MMP-13 expression.

**Conclusion:**

Botanical metabolites derived from the genus *Epimedium* demonstrate pharmacological potential in Murine models of OA. The underlying mechanisms likely involve attenuating the production of systemic and local inflammatory factors and ameliorating cartilage damage.

## 1 Introduction

Osteoarthritis (OA) is a highly prevalent degenerative joint disease worldwide and a leading cause of pain (Xie et al., 2025), functional impairment, and disability, particularly in the middle-aged and elderly population (Chen et al., 2025). Consequently, OA imposes a substantial medical and economic burden on society (Gupta et al., 2005). Traditionally, OA was perceived primarily as a form of “physical wear and tear” of articular cartilage resulting from mechanical stress. However, recent medical advancements have fundamentally shifted this paradigm, redefining OA as a multifactorial “whole-joint disease” driven by metabolic dysregulation and aberrant immune responses (Man and Mologhianu, 2014). The pathogenesis of OA encompasses more than the degradation of the chondrocyte extracellular matrix (ECM); it is integrally linked with synovial macrophage polarization dysregulation, mitochondrial dysfunction, and aberrant subchondral bone remodeling (He et al., 2020). Current clinical management strategies for OA primarily focus on alleviating pain and improving joint function, including non-steroidal anti-inflammatory drugs (NSAIDs) and intra-articular injections of corticosteroids or hyaluronic acid. However, these conventional therapies offer only short-term symptomatic relief without fundamentally delaying or reversing cartilage degeneration (Testa et al., 2021). Moreover, their long-term use is associated with potential adverse effects, including gastrointestinal, cardiovascular, and renal complications (Zhang et al., 2016). Therefore, there is an urgent need to develop disease-modifying osteoarthritis drugs (DMOADs) that can effectively intervene in the disease process and exert chondroprotective effects (Muthu et al., 2023).

The genus *Epimedium* (Berberidaceae) is widely used in Traditional Chinese Medicine (TCM) to treat “Bi syndrome”—a condition characterized by joint pain and stiffness that closely corresponds to the clinical presentation of OA (Tong et al., 2023). The pharmacological effects of *Epimedium* primarily stem from its specific botanical metabolites, predominantly prenylated flavonoids such as icariin (ICA; chemical name: 8-prenyl-kaempferol 4′-methyl ether 3-O-α-L-rhamnopyranoside-7-O-β-D-glucopyranoside). Structurally, icariin is a kaempferol derivative characterized by an 8-prenyl group, which confers enhanced lipophilicity and a potential binding affinity to the osteoarticular microenvironment (Mukai, 2018; Zhang et al., 2022; Tong et al., 2023). Recent evidence indicates that ICA possesses broad-spectrum antioxidant and anti-inflammatory properties, as demonstrated across various cardiovascular, neurological, and metabolic disease models (Singh and Singh, 2025). While previous experimental evidence highlights its ability to suppress inflammatory cascades and extracellular matrix degradation, the molecular interactions between these known botanical metabolites and specific targets on osteoarticular cells require further systematic elucidation. Furthermore, no systematic meta-analysis has yet been undertaken to rigorously evaluate the overall pharmacological effects of *Epimedium*-derived interventions. To address this gap, the present study employs a systematic review and meta-analysis of available literature to objectively assess their impact on key outcome measures in Murine subjects with OA, thereby providing a robust theoretical foundation for future mechanistic investigations and clinical trial design.

## 2 Methods

This systematic review and meta-analysis was conducted and reported in accordance with the Preferred Reporting Items for Systematic Reviews and Meta-Analyses (PRISMA) guidelines (Moher et al., 2009). The study protocol was designed following the SYRCLE’s (Systematic Review Centre for Laboratory animal Experimentation) guidelines (Hooijmans et al., 2014) and was prospectively registered with PROSPERO (Registration No. CRD420251143826).

### 2.1 Literature search

Two authors independently searched the following electronic databases from their inception to October 2025: PubMed, Web of Science, Foreign Medical Sciences Full-Text Database (FMRS), and Embase for English publications; and the China National Knowledge Infrastructure (CNKI), Wanfang Data, and VIP Database for Chinese publications. The search strategy was developed by combining Medical Subject Headings (MeSH) with free-text terms, adapted to the specific syntactic rules of individual databases. The core search concepts encompassed three logical dimensions: the target disease, incorporating terms for osteoarthritis and cartilage degeneration; the applied intervention, specifically targeting *Epimedium* alongside its core botanical metabolites including icariin; and the experimental subjects, strictly limited to Murine subjects. The comprehensive search strings are provided in Supplementary Appendix 1.

### 2.2 Inclusion and exclusion criteria

Study selection strictly adhered to the predefined PICOS (Population, Intervention, Comparison, Outcomes, Study design) framework: (P) Population: Murine subjects with experimentally established OA. (I) Intervention: Studies employing crude extracts of *Epimedium* or its purified active botanical metabolites (primarily icariin and its derivatives) as the sole intervention. (C) Comparison: OA model animals receiving a placebo or an equivalent volume of vehicle. (O) Outcomes: Studies reporting at least one prespecified core quantitative metric: histomorphological scores, serum inflammatory markers, or local intra-articular inflammatory markers. (S) Study design: Randomized, controlled, preclinical *in vivo* animal studies.

The exclusion criteria were (1) non-original articles (e.g., reviews, meta-analyses, conference abstracts): or studies with incomplete data; (2) clinical trials, *in vitro* studies, retrospective studies, case reports, or protocols; (3) studies with unspecified interventions, doses, or durations; (4) studies in which the OA model group received concomitant interventions with known pharmacological effects against OA.

### 2.3 Study selection

After removing duplicates, two reviewers independently screened the titles and abstracts of the retrieved records to exclude irrelevant studies. The full texts of the potentially eligible articles were then assessed for final inclusion. Any disagreements between the reviewers were resolved by consensus or consultation with a third reviewer.

### 2.4 Data extraction

Two reviewers independently extracted the following data from each included study using a standardized form: first author, publication year, method of OA induction, sample size per group, type of *Epimedium* botanical metabolites, intervention details (dose, administration route, duration), and outcome measures (mean and standard deviation [SD]). If a study reported multiple doses, data from the highest dose group were extracted. For data presented graphically, numerical values were extracted using Engauge Digitizer software.

### 2.5 Risk of bias assessment

The risk of bias for each included study was independently assessed by two reviewers using the SYRCLE’s risk of bias tool for animal studies. This tool evaluates ten domains: (1) sequence generation, (2) baseline characteristics, (3) allocation concealment, (4) random housing, (5) blinding of participants and personnel, (6) random outcome assessment, (7) blinding of outcome assessors, (8) incomplete outcome data, (9) selective outcome reporting, and (10) other sources of bias. Each domain was judged as having a “low,” “high,” or “unclear” risk of bias. Discrepancies were resolved through discussion with a third reviewer.

### 2.6 Statistical analysis

All continuous outcomes were expressed as the mean and standard deviation (SD). The standardized mean difference (SMD) was selected as the effect size metric, and Hedges’ g was specifically calculated to effectively correct for the upward overestimation bias associated with small sample sizes common in preclinical studies involving Murine subjects. All data synthesis and statistical analyses were performed using Stata 19.0 and RevMan 5.4 software. Given the inherent potential heterogeneity across preclinical animal experiments—arising from variations in modeling mechanisms, dosages, intervention durations, and commercial sources of ELISA kits—a random-effects model was universally applied for all statistical inferences. For the estimation of between-study variance (τ^2^), the Sidik-Jonkman (SJ) estimator was employed throughout due to its superior robustness in small-sample datasets. Statistical heterogeneity among the included studies was quantitatively evaluated using the I^2^ and H^2^ statistics. To achieve a rigorous scientific balance between controlling the Type I error rate and maintaining adequate statistical power, a conditional application of the Knapp-Hartung (KH) standard error adjustment was implemented: the KH adjustment was applied to the random-effects model only when the number of independent studies included for a specific outcome exceeded 3. To further explore potential sources of significant heterogeneity, univariate subgroup analyses were conducted for the core outcomes based on the OA induction model and the route of administration. Methodological risk of bias was evaluated using the SYRCLE tool for animal studies. Potential publication bias and small-study effects were visually explored using contour-enhanced funnel plots and quantitatively assessed via Egger’s linear regression test. Furthermore, the trim-and-fill method was introduced as a sensitivity analysis to evaluate and verify the robustness of the overall findings regarding pharmacological effects. A two-sided *P*-value of <0.05 was considered statistically significant.

## 3 Results

### 3.1 Study selection

**FIGURE 1:**
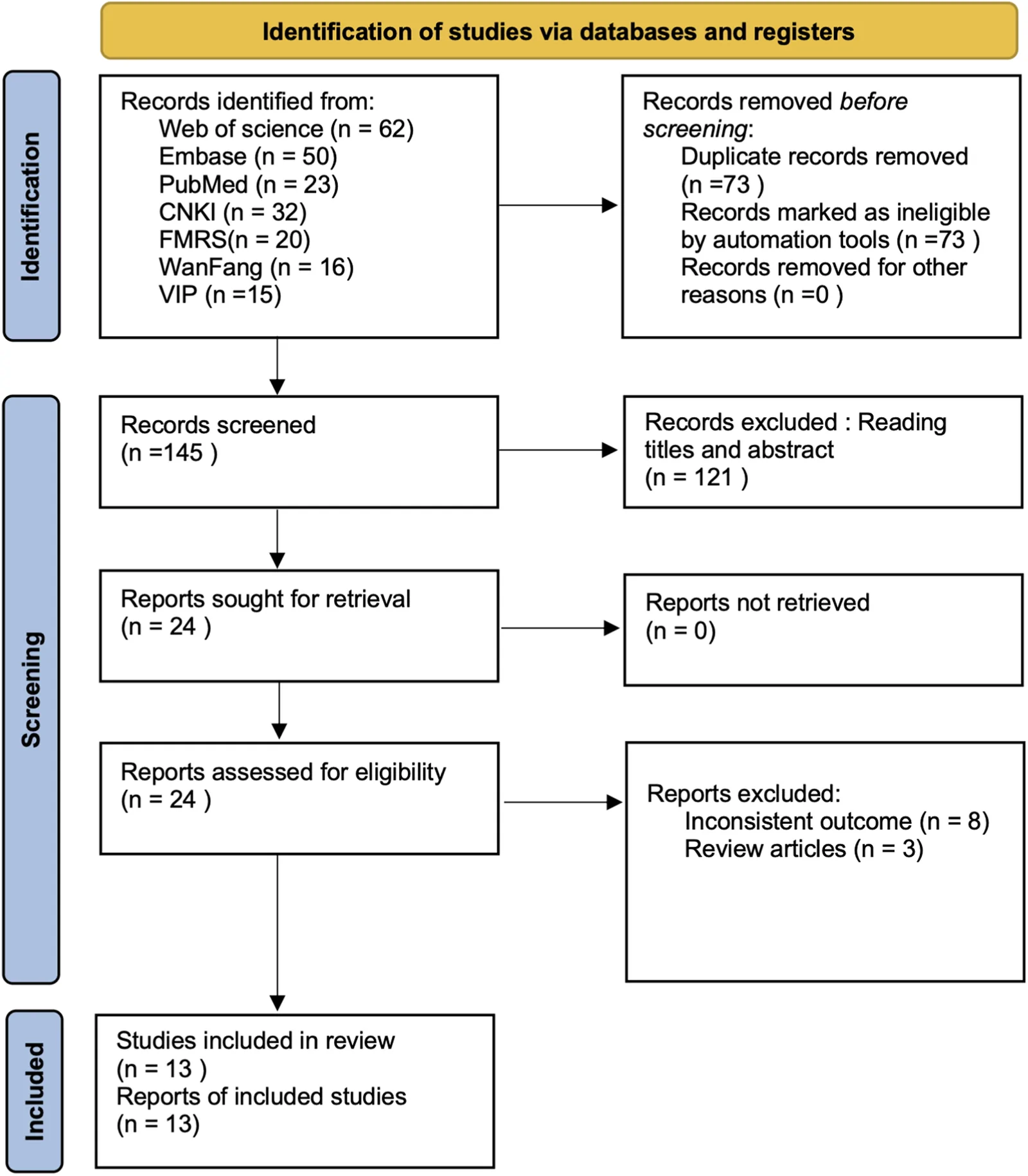
PRISMA flow chart of study selection. PRISMA, preferred reporting items for systematic reviews and meta-analysis.

The initial literature search across all databases yielded a total of 218 records. After 73 duplicate records were removed using Zotero 7.0.16 software, 145 unique articles were screened based on their titles and abstracts. During this stage, 121 articles were excluded as they did not meet the inclusion criteria (e.g., irrelevant topic, incorrect study design, or wrong intervention). Subsequently, the full texts of the remaining 24 articles were retrieved and assessed for eligibility. A further 11 reports were excluded for specific reasons, primarily for not reporting the prespecified outcomes (n = 8) or being non-original research (n = 3). Ultimately, 13 studies fulfilled all criteria and were included in the final systematic review and meta-analysis. A detailed flowchart of this selection process is presented in Figure 1 (Dai, 2022; Ma et al., 2024; Tang et al., 2021; Yu et al., 2023; Zeng et al., 2014; Zu et al., 2019; Feng et al., 2024; Feng et al., 2023; Shi et al., 2021; Wu et al., 2025; Wu et al., 2023; Jin et al., 2020; Gao et al., 2017).

### 3.2 Characteristics of included studies

The 13 included studies were published between 2010 and 2025 and investigated the pharmacological actions of *Epimedium*-related botanical metabolites on OA models in Murine subjects. OA induction methods comprised the Hulth method (n = 5), anterior cruciate ligament transection (ACLT; n = 4), mono-iodoacetate (MIA) injection (n = 3), and collagenase-induced osteoarthritis (CIOA; n = 1). Regarding experimental control design, multiple studies rigorously employed normal saline as the vehicle control for *in vivo* administration, providing a reliable baseline for evaluating pharmacological effects. In accordance with their pharmacological properties, the intervention materials were classified into botanical metabolites (e.g., purified icariin, n = 9), botanical drugs/crude extracts (n = 3), and total flavonoids (n = 1). Regarding preparation characterization, three studies explicitly reported using 50% ethanol as the extraction solvent, whereas one study utilized a water decoction method.

Taxonomic authentication indicated that three studies precisely identified the botanical origin as *Epimedium brevicornu* Maxim. (Berberidaceae). Regarding the characterization of medicinal parts, the source materials in these three studies were all explicitly specified as dried leaves (n = 3). Conversely, the remaining ten studies, which directly utilized commercially procured purified botanical metabolites, lacked specific species-level taxonomic details for their actual experimental materials and failed to report the specific plant organs used. The primary routes of administration included oral gavage (n = 8), intra-articular injection (n = 4), and intraperitoneal injection (n = 1). The baseline characteristics of these included studies are summarized in Table 1.

**TABLE 1:** Characteristics of the included studies.

First author (year)	OA model	Sample size (T/C)	Botanical drug/Plant metabolite (preparation)	Taxonomic validation	Methods of administration	Dosage (T/C)	Duration
Jin et al. (2020)	ACLT	10/10	Total flavonoids of *Epimedium* (50% ethanol extract)	*Epimedium brevicornu* maxim. (Berberidaceae)	Oral gavage	600 mg/(kg·d)/NS	6 weeks
Wu et al. (2023)	Hulth	10/10	*Epimedium* extract (50% ethanol extract)	*Epimedium brevicornu* maxim. (Berberidaceae)	Oral gavage	13.4 mg/(kg·d)/NS	14 weeks
Wu et al. (2025)	Hulth	10/10	*Epimedium* extract (50% ethanol extract)	*Epimedium brevicornu* maxim. (Berberidaceae)	Oral gavage	13.4 mg/(kg·d)/NS	6 weeks
Dai (2022)	CIOA	10/10	*Epimedium* extract (water decoction)	Not specified	Oral gavage	0.75 g/(kg·d)/NS	4 weeks
Gao et al. (2017)	ACLT	10/10	Icariin	Not specified	Oral gavage	10 mg/(kg·d)/NS	8 weeks
Feng et al. (2023)	Hulth	10/10	Icariin	Not specified	Oral gavage	100 mg/(kg·d)/NS	8 weeks
Feng et al. (2024)	ACLT	10/10	Icariin	Not specified	Intra-articular injection	100 mg/(kg·d)/NS	8 weeks
Shi et al. (2021)	MIA	10/10	Icariin	Not specified	Oral gavage	50 mg/(kg·d)/NS	5 weeks
Zu et al. (2019)	MIA	5/5	Icariin	Not specified	Intra-articular injection	20 μM (0.3 mL)/NS	6 weeks
Tang et al. (2021)	Hulth	6/6	Icariin	Not specified	Intraperitoneal injection	80 mg/(kg·d)/NS	4 weeks
Ma et al. (2024)	Hulth	10/10	Icariin	Not specified	Oral gavage	20 mg/(kg·d)/NS	6 weeks
Yu et al. (2023)	MIA	5/5	Icariin	Not specified	Intra-articular injection	50 μg/d/NS	9 weeks
Zeng et al. (2014)	ACLT	10/10	Icariin	Not specified	Intra-articular injection	20 μM (0.3 mL)/NS	10 weeks

ACLT, Anterior Cruciate Ligament Transection; Hulth, Hulth Model; MIA, Mono-iodoacetate-induced Osteoarthritis; CIOA, Collagenase-Induced Osteoarthritis Model; T/C, treatment/control sample size ratio; NS, normal saline.

### 3.3 Methodological risk of bias assessment

**FIGURE 2:**
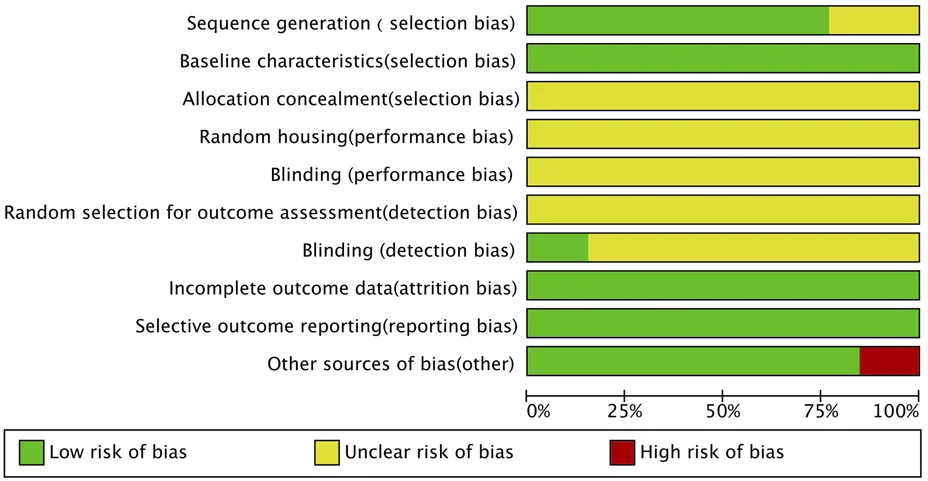
Quality of the included studies.

The methodological quality of the 13 included studies was evaluated using the SYRCLE risk of bias tool for animal studies, with the results depicted in Figure 2. Overall, the majority of the studies lacked crucial information regarding core bias-minimization designs: none of the included studies adequately reported key details on allocation concealment, random housing, and the blinding of personnel and outcome assessors, thereby presenting a high or unclear risk of bias. Specifically, only three studies clearly described their methods for random sequence generation. Conversely, all 13 studies were judged to have a low risk of bias concerning incomplete outcome data and selective outcome reporting. The absence of these core methodological details underscores that caution is warranted when interpreting the certainty of the preclinical proof-of-concept evidence for this botanical drug.

### 3.4 Meta-analysis

#### 3.4.1 Histological assessment of cartilage degeneration

**FIGURE 3:**
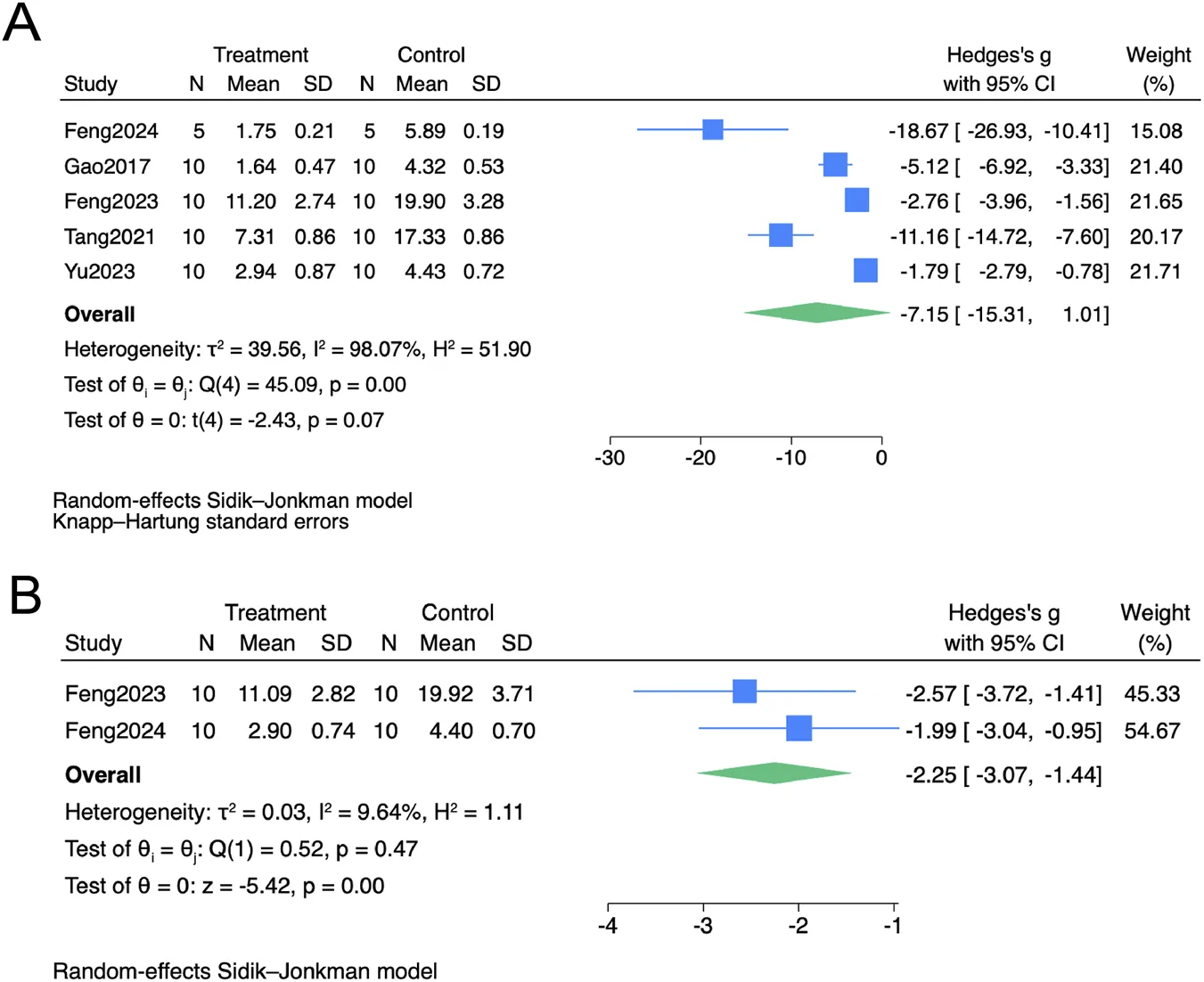
Forest plot between the *Epimedium* group and the control group. **(A)** OARSI. **(B)** Pelletier.

The pooled analysis of five studies reporting OARSI scores revealed that, relative to the control group, the intervention group exhibited a clear reducing trend in cartilage degeneration, which nonetheless failed to reach the threshold of statistical significance (SMD = −7.15, 95% CI: 15.31 to 1.01, P = 0.07; Figure 3A). Similarly, accumulated data from two studies utilizing the Pelletier score demonstrated a significant chondroprotective pharmacological effect (SMD = −2.25, 95% CI: 3.07 to −1.44, P < 0.001; Figure 3B).

#### 3.4.2 Effects on Serum Inflammatory Markers

**FIGURE 4:**
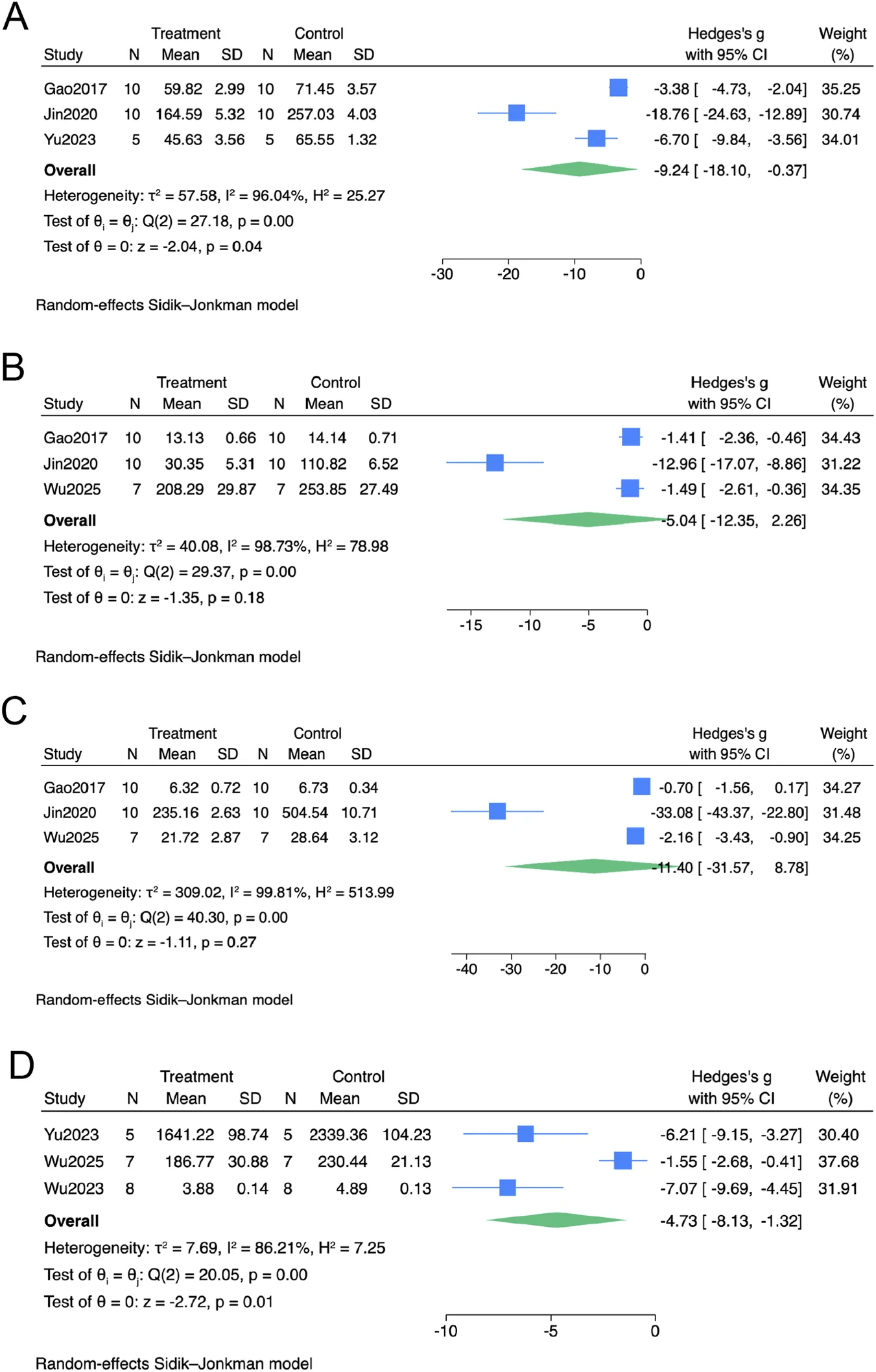
Forest plot comparing Effects on Serum Inflammatory Markers between the *Epimedium* group and the control group. **(A)** IL-6. **(B)** TNF-α. **(C)** IL-1β. **(D)** MMP-13.

Pooled data from three studies indicated that *Epimedium* intervention elicited significant reductions in serum IL-6 (SMD = −9.24, 95% CI: 18.10 to −0.37, P = 0.04; Figure 4A) and MMP-13 levels (SMD = −4.73, 95% CI: 8.13 to −1.32, P = 0.01; Figure 4D). Furthermore, although numerical downward trends were observed in serum TNF-α (SMD = −5.04, 95% CI: 12.35 to 2.26, P = 0.18; Figure 4B) and IL-1β (SMD = −11.40, 95% CI: 31.57 to 8.78, P = 0.27; Figure 4C), these inter-group differences did not achieve statistical significance.

#### 3.4.3 Effects on pain-related behaviors

**FIGURE 5:**
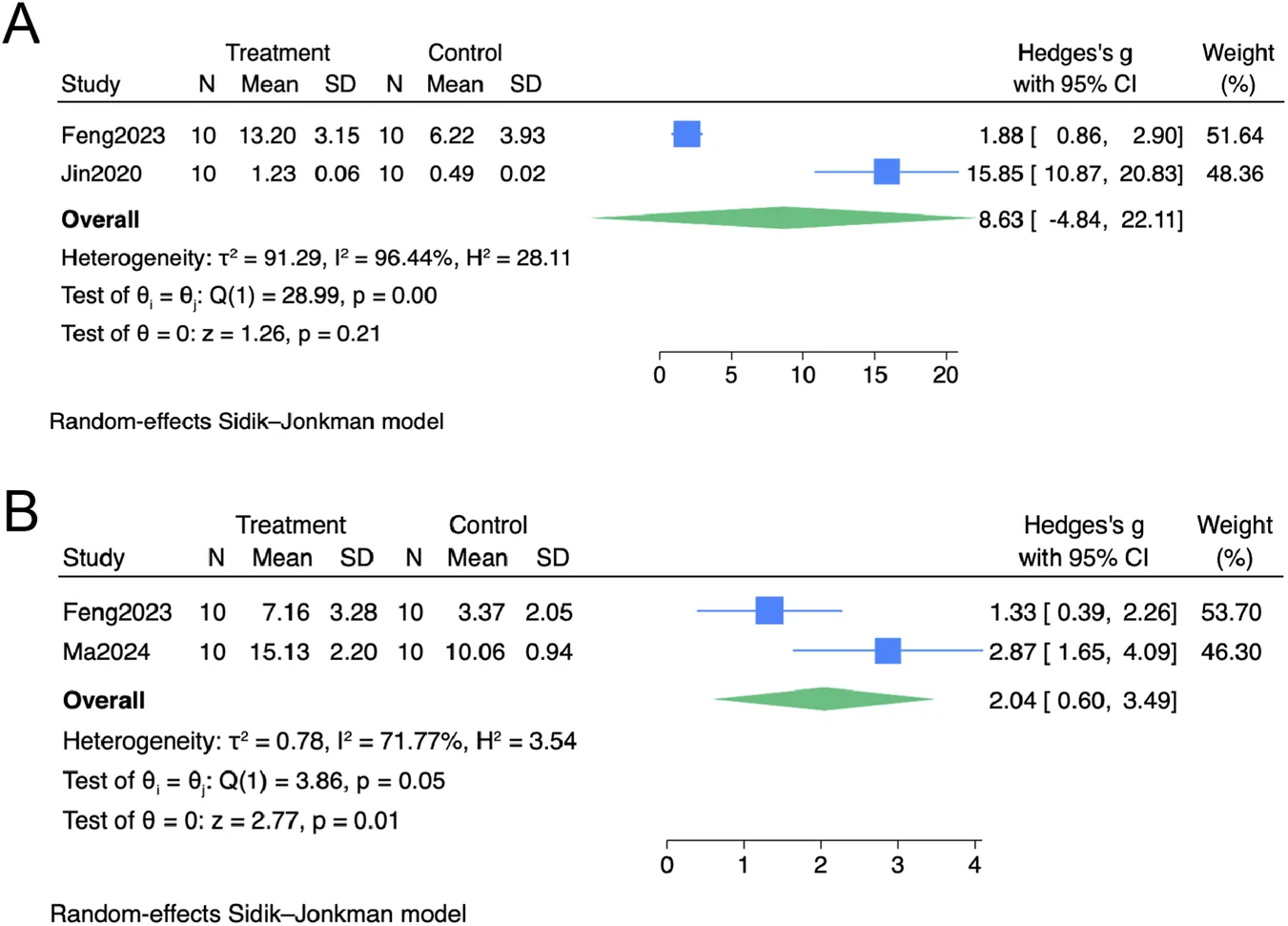
Forest plot between the *Epimedium* group and the control group. **(A)** PWT. **(B)** PWL.

Analysis of data from three studies revealed an increase in the mechanical paw withdrawal threshold (PWT) following the intervention; however, this analgesic pharmacological effect did not reach statistical significance (SMD = 8.63, 95% CI: 4.84 to 22.11, P = 0.21; Figure 5A). Additionally, pooled results from two studies demonstrated a significant increase in paw withdrawal latency (PWL) to thermal stimuli (SMD = 2.04, 95% CI: 0.60 to 3.49, P = 0.01; Figure 5B). Behavioral metrics such as PWT and PWL rely heavily on subjective observer assessment. Given that the included studies were predominantly rated as having an ‘unclear’ or ‘high risk’ of bias in the domain of ‘blinding of outcome assessment’, we must explicitly acknowledge this limitation when interpreting the analgesic pharmacological effects and remain vigilant regarding the potential overestimation of the effect sizes.

#### 3.4.4 Effects on Intra-articular protein expression

**FIGURE 6:**
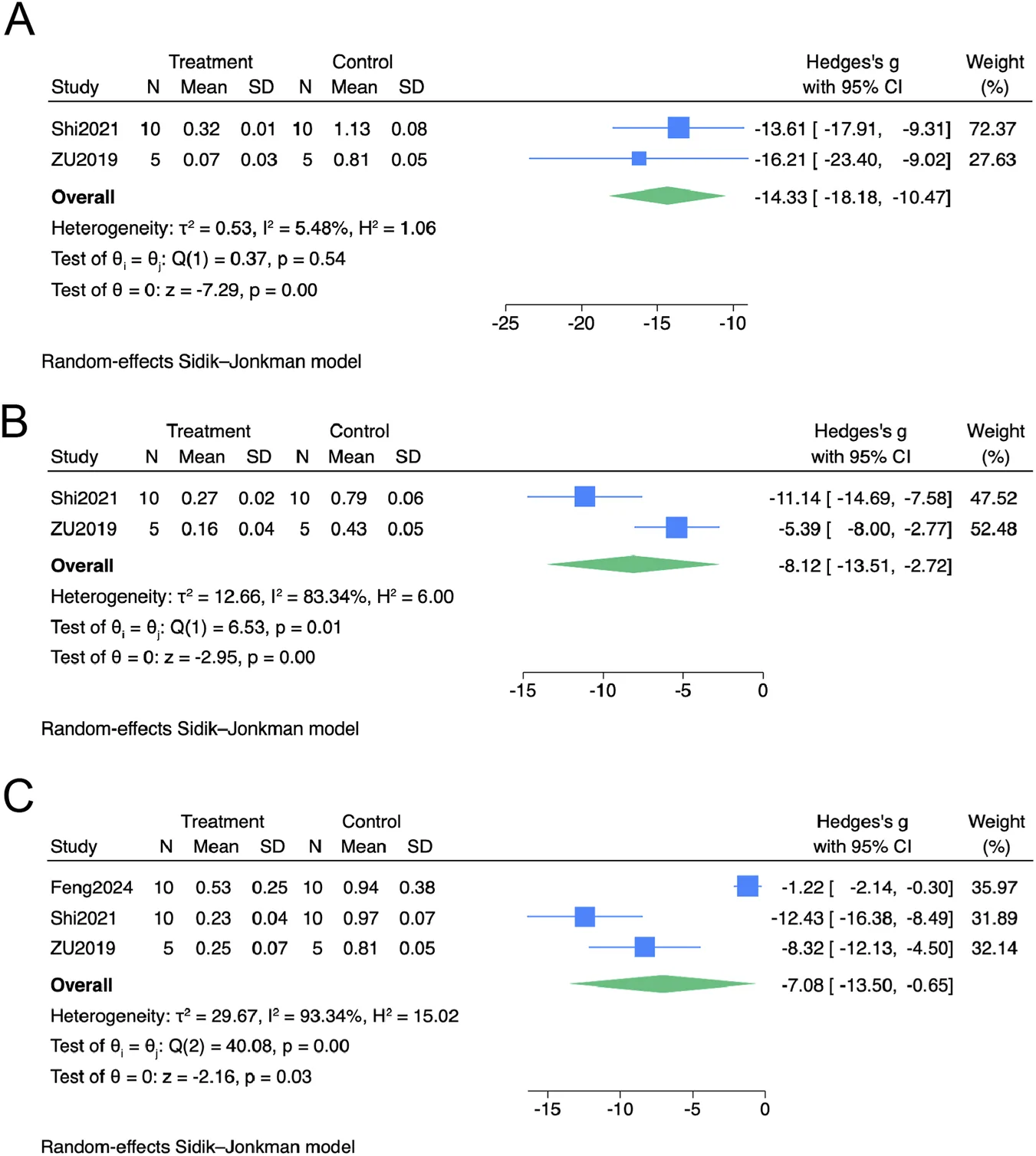
Forest plot comparing Effects on Intra-articular Protein Expression between the *Epimedium* group and the control group. **(A)** MMP-13. **(B)** IL-18. **(C)** IL-1β.

**FIGURE 7:**
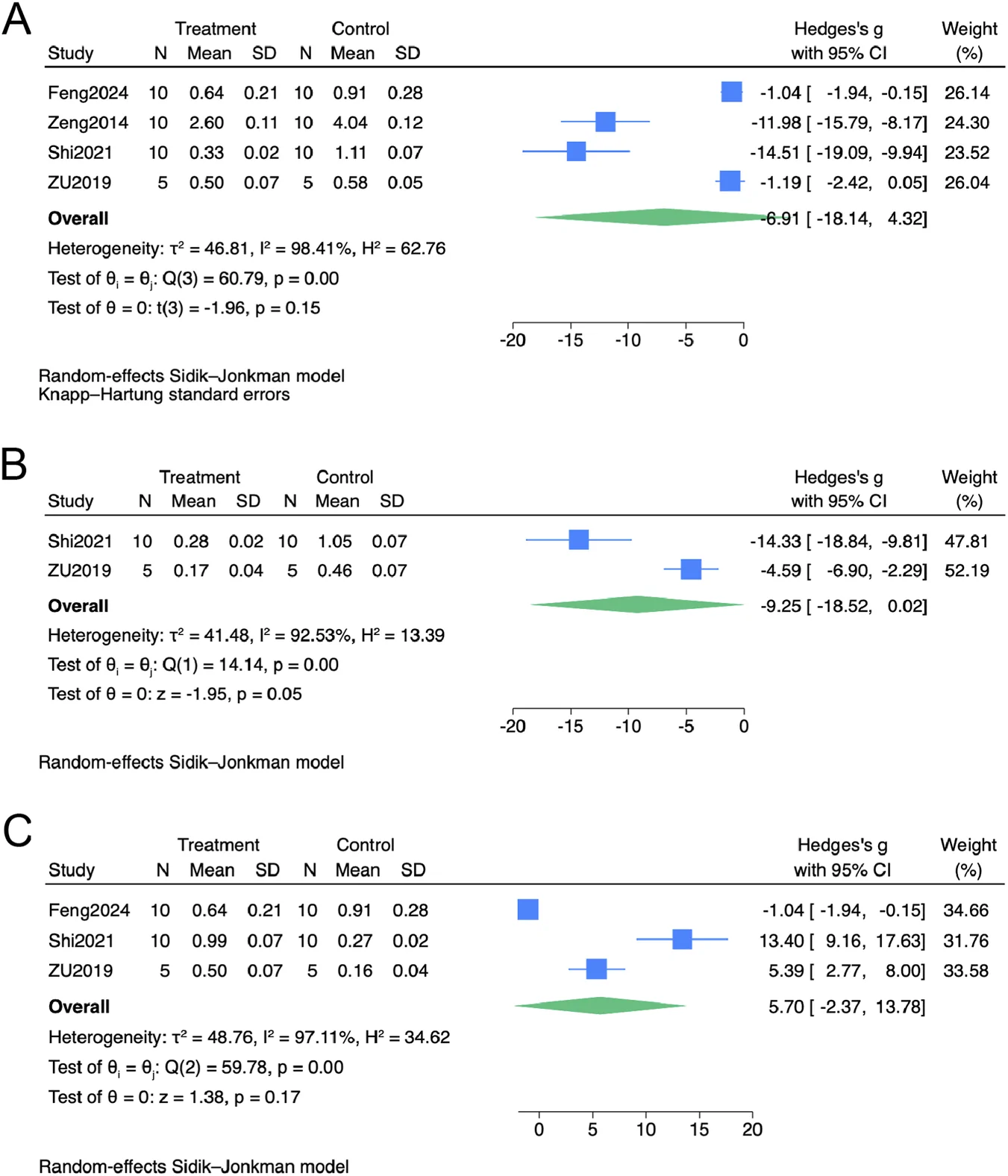
Forest plot comparing Effects on Intra-articular Protein Expression between the *Epimedium* group and the control group. **(A)** MMP-1. **(B)** NLRP3. **(C)** Type II Collagen.

*Epimedium* intervention modulated the expression of catabolic and inflammatory proteins within the joint tissues. Specifically, data synthesized from two studies demonstrated a significant downregulation of IL-18 (SMD = −14.33, 95% CI: 18.18 to −10.47, P < 0.001; Figure 6A) and IL-1β (SMD = −8.12, 95% CI: 13.51 to −2.72, P < 0.01; Figure 6B), while pooled results from three studies revealed significantly reduced levels of MMP-1 (SMD = −7.08, 95% CI: 13.50 to −0.65, P = 0.03; Figure 6C). Concurrently, non-significant downward trends were observed in an analysis of four studies for MMP-13 (SMD = −6.91, 95% CI: 18.14 to 4.32, P = 0.15; Figure 7A) and two studies for NLRP3 (SMD = −9.25, 95% CI: 18.52 to 0.02, P = 0.05; Figure 7B), whereas data from three studies exhibited a non-significant upward trend in Type II collagen expression (SMD = 5.70, 95% CI: 2.37 to 13.78, P = 0.17; Figure 7C).

#### 3.4.5 Effects on gene expression in chondrocytes

**FIGURE 8:**
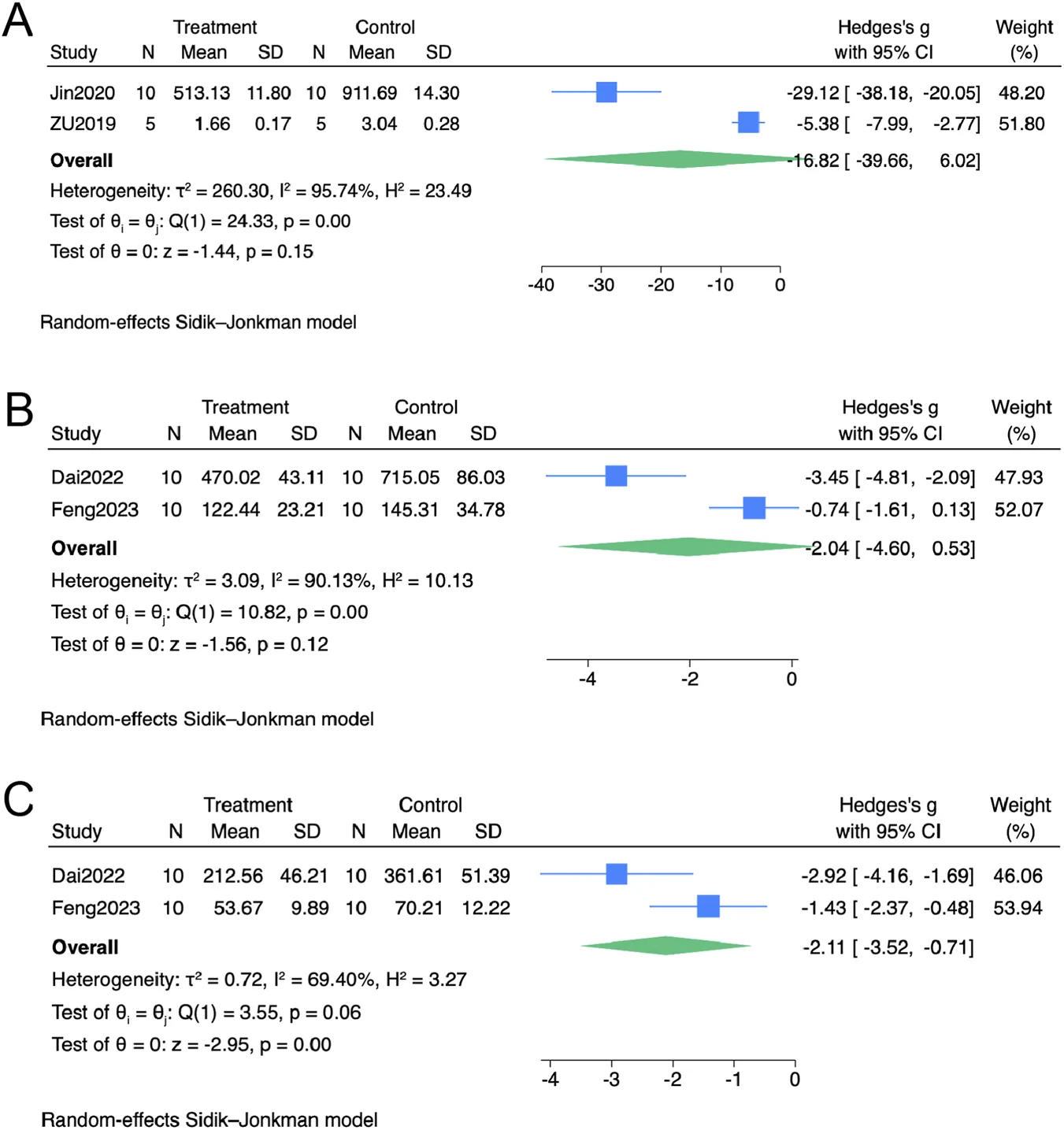
Forest plot between the *Epimedium* group and the control group. **(A)** IL-1β mRNA expression in chondrocytes. **(B)** Effects on Inflammatory Markers in Synovial Fluid of TNF-α. **(C)** Effects on Inflammatory Markers in Synovial Fluid of IL-1β.

The pooled analysis of two studies assessing IL-1β mRNA expression in chondrocytes revealed a non-significant downward trend following the intervention (SMD = −16.82, 95% CI: 39.66 to 6.02, P = 0.15; Figure 8A).

#### 3.4.6 Effects on Inflammatory Markers in Synovial Fluid

An analysis of inflammatory markers in synovial fluid from two studies revealed that *Epimedium* intervention elicited a non-significant downward trend in TNF-α levels (SMD = −2.04, 95% CI: 4.60 to 0.53, P = 0.12; Figure 8B), while exerting a significant downregulatory pharmacological effect on IL-1β levels (SMD = −2.11, 95% CI: 3.52 to −0.71, P < 0.01; Figure 8C).

### 3.5 Subgroup analysis

**FIGURE 9:**
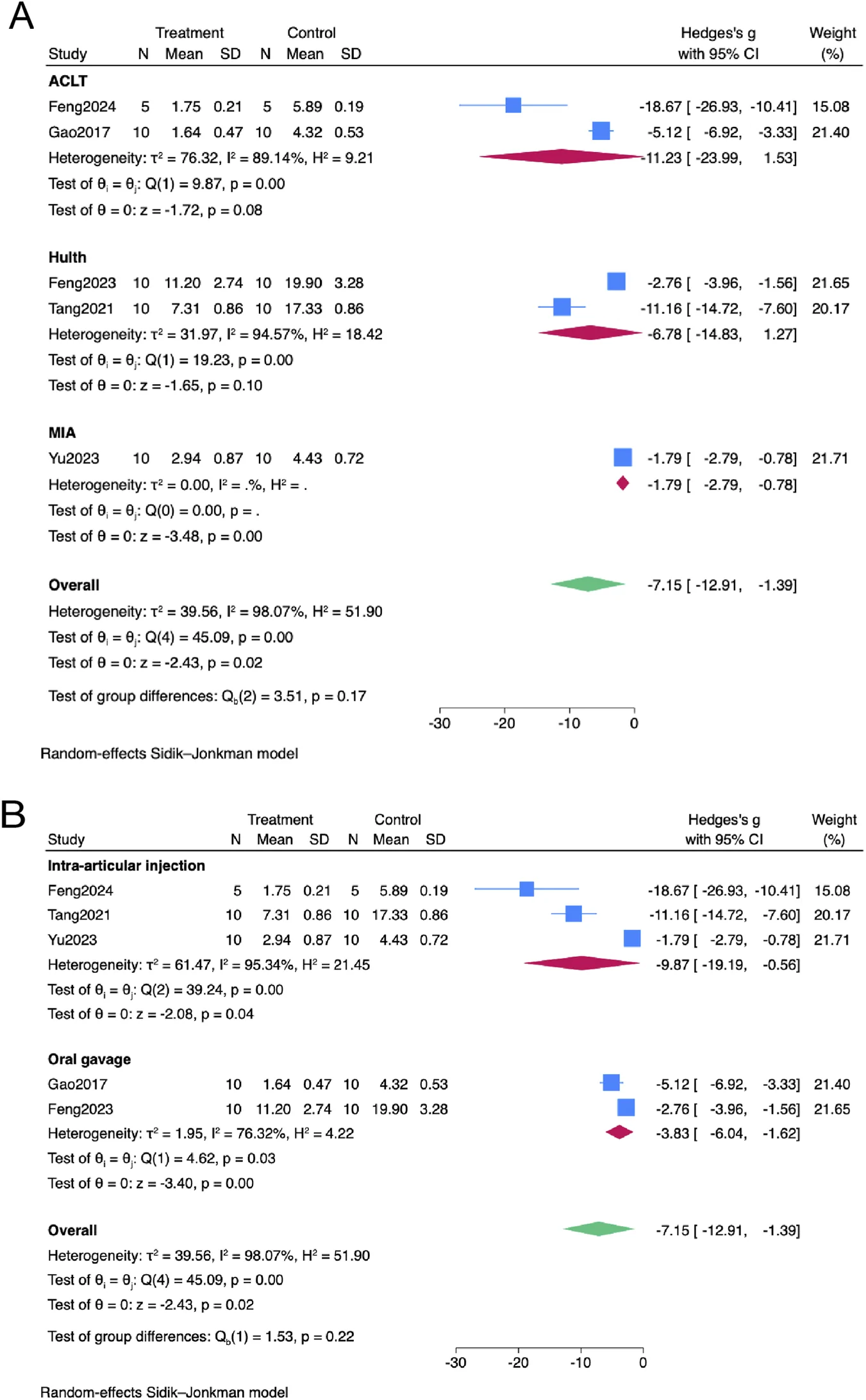
Forest plots of subgroup analyses for the OARSI score. **(A)** Subgroup analysis based on OA induction models. **(B)** Subgroup analysis based on the route of administration.

**FIGURE 10:**
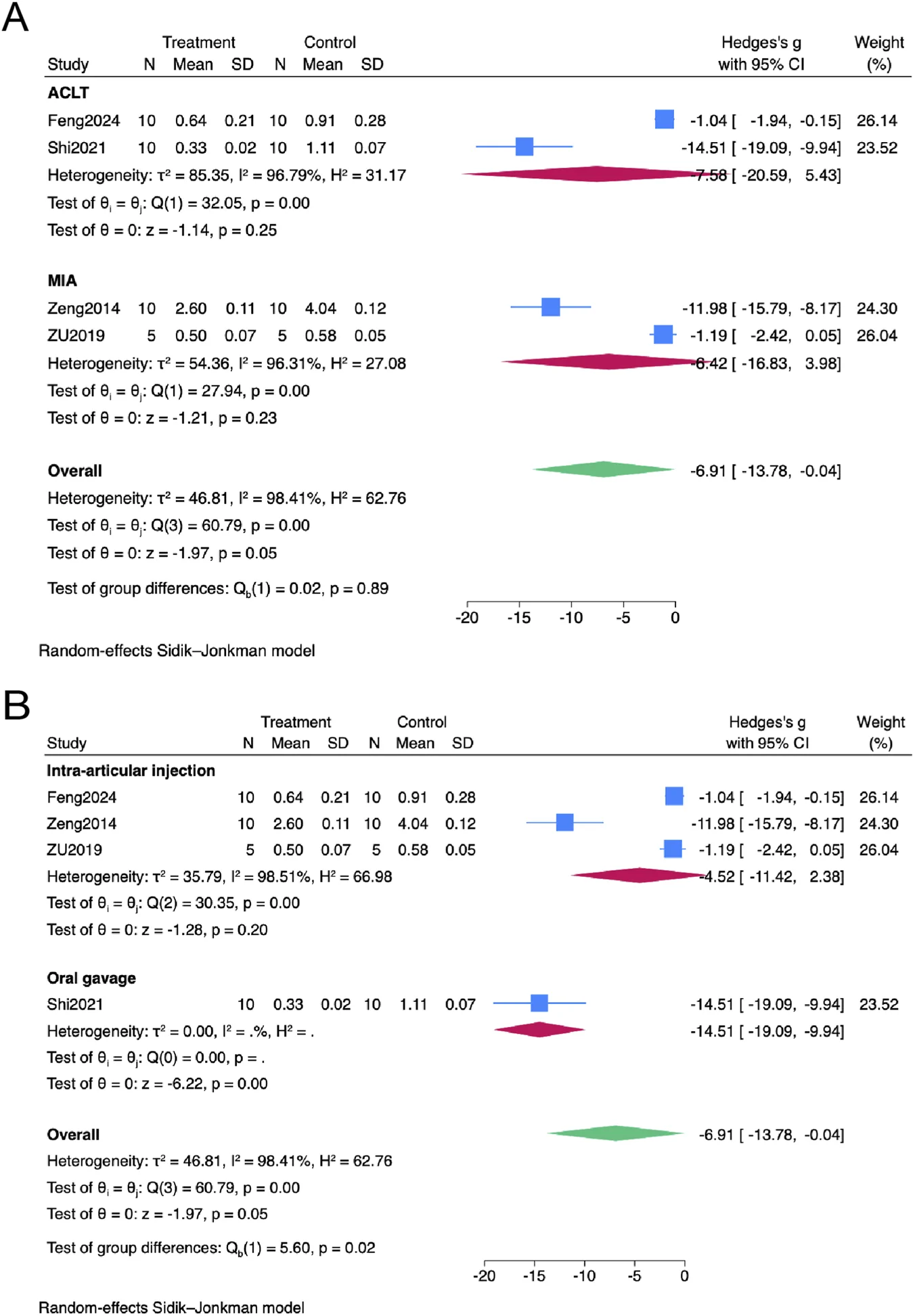
Forest plots of subgroup analyses for intra-articular MMP-13 expression. **(A)** Subgroup analysis based on OA induction models. **(B)** Subgroup analysis based on the route of administration.

To explore the sources of significant heterogeneity among the included studies and evaluate the impact of varying experimental conditions on the pharmacological effects of *Epimedium* formulations, univariate subgroup analyses were conducted for the core outcomes (including the OARSI score and intra-articular MMP-13 protein expression). These analyses universally applied the Sidik-Jonkman variance estimator to assess potential confounding factors, such as the murine OA induction model and the route of administration, that might contribute to the observed high heterogeneity. As illustrated in Figure 9, the choice of OA induction models (ACLT, Hulth, and MIA) did not emerge as a primary source of heterogeneity for either the OARSI score or MMP-13 expression (test for subgroup differences: P = 0.17 and P = 0.89, respectively). Additionally, the route of administration failed to account for the substantial heterogeneity observed in the OARSI score (P = 0.22). However, in the analysis targeting intra-articular MMP-13 expression (Figure 10), the route of administration was identified as a significant contributor to the prominent heterogeneity (P = 0.02). Notably, the oral gavage group exhibited a significantly more pronounced downregulatory pharmacological effect on MMP-13 expression compared to the intra-articular injection group (SMD = −14.51 vs. −4.52).

### 3.6 Publication bias and small-study effects analysis

**FIGURE 11:**
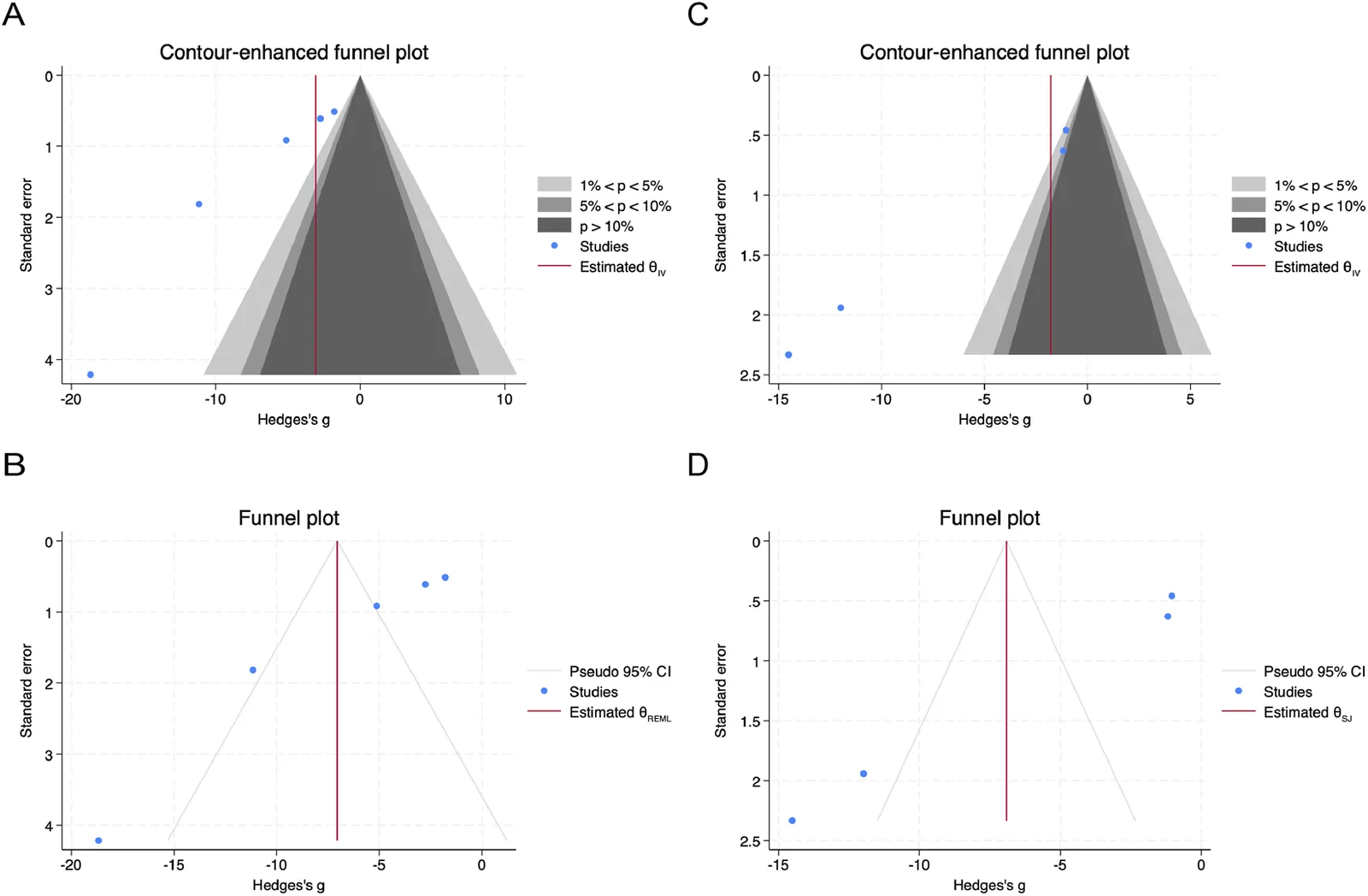
Analysis of publication bias and small-study effects for the core outcomes. **(A)** Contour-enhanced funnel plot for the OARSI score. **(B)** Trim-and-fill funnel plot for the OARSI score. **(C)** Contour-enhanced funnel plot for intra-articular MMP-13 expression. **(D)** Trim-and-fill funnel plot for intra-articular MMP-13 expression.

Potential small-study effects for the two core outcomes (the OARSI score and intra-articular MMP-13 expression) were quantitatively assessed using Egger’s linear regression test. The results revealed highly significant asymmetry in the traditional funnel plots for both metrics (Egger’s test: P < 0.001), suggesting the potential presence of publication bias within these datasets. To further elucidate the underlying sources of this asymmetry, contour-enhanced funnel plots were generated for in-depth profiling. For the OARSI score (Figure 11A), all five studies located to the left of the central axis fell entirely within the highly significant (unshaded) peripheral regions (P < 0.01), whereas a conspicuous absence of data points was observed in the dark-shaded regions corresponding to statistical non-significance (P > 0.10). For intra-articular MMP-13 expression (Figure 11C), although most off-axis studies (three studies) similarly fell into the highly significant peripheral regions, one study was scattered within the lightly shaded boundary zone near the central axis. Taken together, while the scattering patterns of the two plots differ slightly, their core features collectively suggest that the funnel plot asymmetry does not primarily stem from the non-publication of conventionally negative or non-significant studies (i.e., selective publication bias). Instead, it is more likely driven by substantial positive pharmacological effects induced by the specific experimental design characteristics of studies with small sample sizes. To verify the robustness of the final pooled data, sensitivity analyses were subsequently performed for both the OARSI score and MMP-13 expression utilizing the trim-and-fill method (Figures 11B,D). Within the datasets of these two independent core outcomes, the algorithm did not identify any missing studies requiring imputation (the number of imputed studies was zero). Consequently, the pooled pharmacological effect sizes and their 95% confidence intervals remained identical before and after bias correction. Nevertheless, given that the substantial heterogeneity and methodological limitations (such as the pervasive lack of blinding) inherent in preclinical *in vivo* experiments may inflate positive outcomes to some extent, the magnitude of the pharmacological effects ultimately demonstrated in OA murine models must still be interpreted with considerable caution.

### 3.7 GRADE assessment

The GRADE (Grading of Recommendations Assessment, Development and Evaluation) approach was formally utilized to evaluate the certainty of the preclinical evidence for each primary outcome measure. As illustrated in the Summary of Findings table in Supplementary Appendix 2, the certainty of evidence across the 17 evaluated outcomes was categorized as moderate (n = 5), low (n = 7), and very low (n = 5). Consequently, the overall certainty of evidence for the outcome metrics in this study was deemed low-to-moderate. The comprehensive GRADE assessment revealed that the primary drivers for evidence downgrading were the high risk of bias across most included studies (pervasive deficiencies in allocation concealment and outcome assessor blinding) and serious statistical inconsistency observed in several core indicators.

### 3.8 *In silico* molecular target interactions

To systematically contextualize the specific botanical metabolites driving the observed pharmacological outcomes, the core *in silico* molecular targets of icariin—the primary active prenylated flavonoid derived from *Epimedium*—in OA are classified and summarized in Table 2. Network pharmacology and molecular docking studies indicate that icariin interacts with an interconnected network of OA-specific targets. These targets span across relevant functional modules, including matrix degradation (e.g., MMP-1, MMP-3, MMP-13), inflammatory cascades (e.g., TNF, IL-6, IL-1β, PTGS2), and intracellular signaling hubs (e.g., AKT1, RELA, PIK3CD) (Zhang et al., 2022; Gu et al., 2023; Tong et al., 2025). Furthermore, while current OA-specific computational models predominantly focus on icariin, it is noteworthy that other structurally related constituents within *Epimedium* botanical metabolites (such as epimedin A, B, and C) demonstrate pharmacological potential in modulating systemic inflammatory and apoptotic networks. For instance, recent network pharmacology and molecular docking studies suggest that in various tissue and disease models—including cisplatin-induced intestinal injury and liver cancer—epimedin A, B, and C can bind to conserved inflammatory and apoptotic signaling hubs, such as NF-κB, TNF-α, p53, and PTGS2 (Xia et al., 2022; Liu Y. M. et al., 2023). This further suggests that the total *Epimedium* extract may exert its anti-inflammatory pharmacological effects within the osteoarticular microenvironment via a multi-component targeting mechanism.

**TABLE 2:** Summary of the core *in silico* molecular targets of Icariin in osteoarthritis based on network pharmacology.

Target classification	Key in silico targets	References
Matrix metalloproteinases	MMP-1, MMP-3, MMP-13	Tong et al., 2025, Gu et al. (2023)
Inflammatory cytokines and mediators	IL-6, IL-1β, TNF, PTGS2 (COX-2)	Tong et al., 2025, Gu et al., 2023, Zhang J. et al. (2022)
Kinases & transcription factors	AKT1, RELA (NF-κB), PIK3CD, MAPK14, MYC, FOS, CCND1	Gu et al., 2023, Zhang J. et al. (2022)
Growth factors and receptors	IGF1, EGFR, ESR1	Gu et al., 2023, Zhang J. et al. (2022)

**FIGURE 12:**
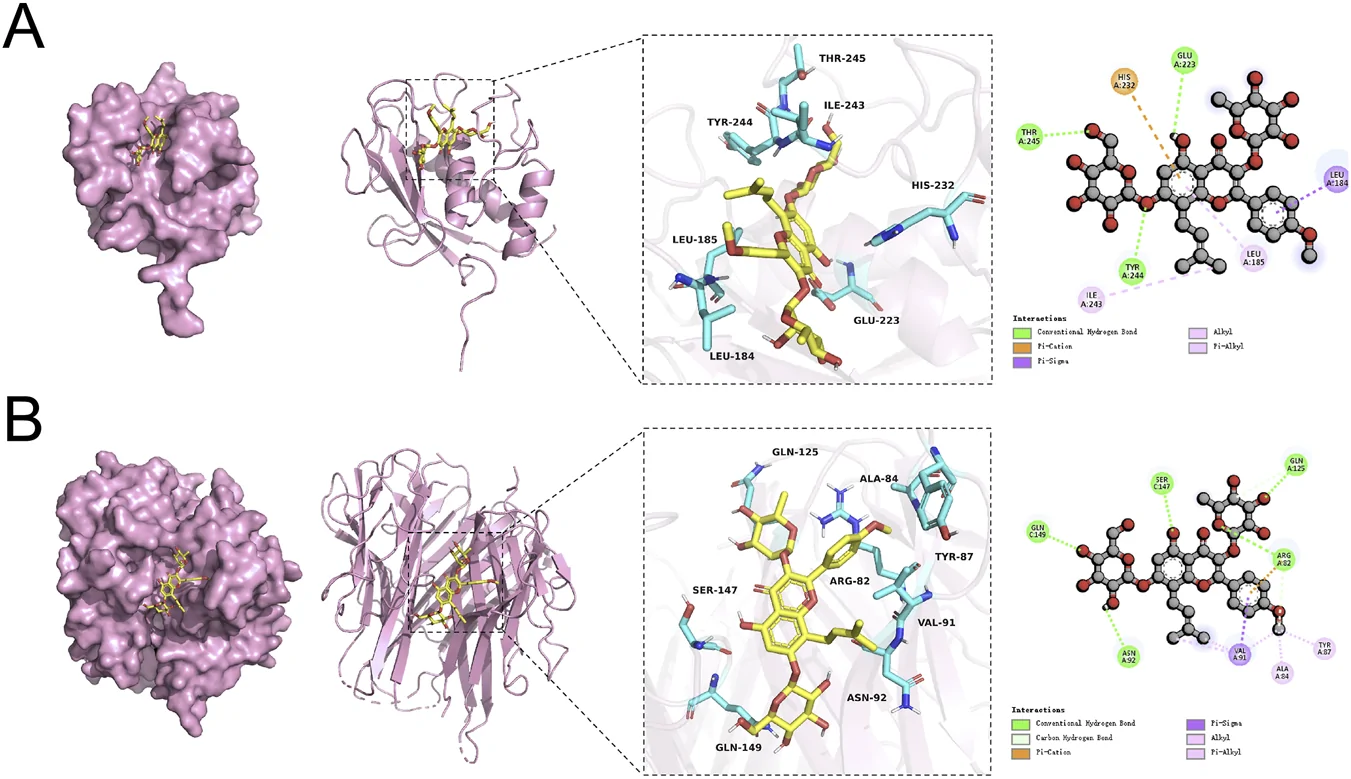
Molecular docking simulations of icariin with core targets. **(A)** Binding mode with MMP-13. **(B)** Binding mode with TNF-α.

Additionally, to establish a structural correlation between these active botanical metabolites and the phenotypic outcomes observed in the present meta-analysis, *in silico* molecular docking simulations were conducted. Specifically, semi-flexible docking was executed using AutoDock Vina 1.2.7 (Eberhardt et al., 2021; Trott and Olson, 2010), targeting active pockets predicted by Cyscore (Cao and Li, 2014). Structural preparation and the subsequent 3D and 2D visualizations were facilitated by ChemDraw 20.0, AutoDock Tools 1.5.7, PyMOL 3.0.3, and Discovery Studio. Given the attenuation of inflammatory mediators and cartilage degradation observed in the quantitative synthesis, TNF-α (PDB: 6OOY) was selected as the upstream inflammatory initiator and MMP-13 (PDB: 1XUD) as the downstream cartilage-degrading effector for docking validation. As illustrated in Figure 12, icariin exhibited spatial binding within the active pockets of both targets. Specifically, icariin formed hydrogen-bonding interactions with the amino acid residues Thr245, His223, and Tyr244 within the catalytic domain of MMP-13 (Figure 12A), alongside additional hydrophobic interactions, yielding a binding affinity of −7.795 kcal/mol. Concurrently, icariin was docked into the trimeric interface of TNF-α (Figure 12B), interacting with residues Asn92, Gln149, Ser147, Gln125, and Arg82 via hydrogen bonds, resulting in a binding affinity of −7.227 kcal/mol. These dual-target docking results offer micro-level visual evidence suggesting that *Epimedium* botanical metabolites may exert their chondroprotective and anti-inflammatory effects by modulating upstream cytokine signaling and downstream matrix metalloproteinase activity.

## 4 Discussion

This meta-analysis systematically evaluated the pharmacological effects of *Epimedium* and its specific botanical metabolites in models involving Murine subjects with osteoarthritis. The ACLT/Hulth and MIA induction models utilized herein demonstrate high pathological fidelity and translational value for validating anti-inflammatory and chondroprotective pathways. Specifically, the ACLT/Hulth model disrupts joint biomechanical homeostasis, successfully recapitulating the chronic pathological cascade of abnormal mechanical stress, synovial immune activation, and the subsequent release of catabolic enzymes seen in human post-traumatic osteoarthritis (PTOA) (Kuyinu et al., 2016; Scanzello and Goldring, 2012). Conversely, the MIA model, by selectively disrupting chondrocyte metabolism and matrix integrity, effectively mimics the complex pain sensitization and severe localized inflammatory microenvironment characteristic of advanced OA, serving as a reliable platform to characterize analgesic and core anti-inflammatory pharmacological effects (Guingamp et al., 1997; Sousa-Valente and Brain, 2019). These two etiologically complementary models collectively establish a rigorous biological foundation for evaluating the multidimensional anti-OA pharmacological actions of these botanical interventions.

Regarding macro-histological damage, the OARSI grading system is an internationally standardized semi-quantitative histological framework that evaluates the global severity of cartilage destruction by scoring the grade and stage of matrix degradation, chondrocyte necrosis, and erosion (Gerwin et al., 2010; Glasson et al., 2010). In contrast, the Pelletier score focuses on quantifying superficial fibrillation, vertical clefts, and localized cell/matrix loss, thereby systematically reflecting the depth and breadth of cartilage degeneration (Pelletier et al., 1983; Pelletier and Martel-Pelletier, 1989). In our assessment, the OARSI score exhibited a favorable mitigating trend, whereas the Pelletier score demonstrated a statistically significant reduction following intervention. These findings collectively underscore that these botanical metabolites hold potential for chondroprotective pharmacological effects, effectively retarding structural progression such as cartilage erosion and the loss of joint integrity.

At the micro-environmental level, these botanical metabolites effectively ameliorate the pathological imbalance between anabolism and catabolism within the extracellular matrix (ECM). MMP-13 is a primary enzyme responsible for degrading Type II collagen, which is the core structural component of the cartilage matrix (Eyre, 2001; Hu and Ecker, 2021). The observed downregulatory trend in intra-articular MMP-13 expression and the upregulation of Type II collagen in our pooled analysis strongly align with these mechanisms. This dual regulatory action is critical, as it suggests that the intervention can shift the balance from a catabolic state, which predominates in OA, towards one of matrix homeostasis and repair.

Contemporary research conceptualizes OA as a “whole-joint” pathology driven by severe synovial inflammation. Within this framework, macrophage polarization dysregulation—specifically M1-type overactivation—serves as a primary driver of synovitis and cartilage erosion (Wen and Liu, 2025). Emerging immunological evidence indicates that these specific botanical metabolites possess distinctive macrophage-targeting immunomodulatory properties, facilitating a phenotypic switch from the pro-inflammatory M1 to the anti-inflammatory/pro-resolving M2 phenotype (Gao et al., 2026; Yan et al., 2024). Mechanistically, they are recognized for their pleiotropic nature (Hu et al., 2025), inhibiting the activation of p38 and JNK within the MAPK pathway and blocking Wnt/β-catenin signaling, both of which lead to the downregulation of MMP expression and a reduction in cartilage matrix degradation (Zeng et al., 2010; Zheng et al., 2025; Liu et al., 2010). Regarding intracellular homeostasis, they promote chondrocyte autophagy as a critical cytoprotective mechanism (Liu Z. et al., 2023) via the modulation of the PI3K/AKT/mTOR signaling pathway (Verma et al., 2022), and activate the Nrf2/ARE antioxidant pathway to scavenge reactive oxygen species and inhibit oxidative stress-induced apoptosis (Charlier et al., 2016; Zuo et al., 2019). Furthermore, pyroptosis has emerged as a critical mediator of inflammatory progression in OA (An et al., 2020). These botanical metabolites can interrupt the inflammatory amplification cascade by downregulating the NF-kappaB signaling pathway and inhibiting NLRP3 inflammasome-mediated pyroptosis, thereby reducing the Caspase-1-dependent release of IL-1β and IL-18 (Mi et al., 2018; Zu et al., 2019). The significant reduction in IL-1β and IL-18 levels observed in our meta-analysis also robustly supports this mechanistic pathway. At the molecular level, the unique architecture of these metabolites enhances cellular permeability (Mukai, 2018) and allows them to competitively occupy the catalytic sites of matrix metalloproteinases (Zhang et al., 2022). Concurrently, these pleiotropic mechanisms also suppress synovial fibrosis and improve vascular endothelial function, facilitating overall cartilage regeneration (Zhang et al., 2026; Wu et al., 2024).

Pain represents the predominant clinical hallmark of patients with OA (Yu et al., 2022). Our evaluation of behavioral metrics revealed that the intervention significantly prolonged thermal paw withdrawal latency (PWL) and exhibited a favorable trend toward elevating the mechanical paw withdrawal threshold (PWT), supporting its potential antinociceptive pharmacological effects. This analgesic effect likely results from both the suppression of local inflammation and the attenuation of neuropathic pain, as mechanistic evidence suggests that these active botanical metabolites reduce pain-related nerve fiber density in subchondral bone via the TRPV1-mediated pathway (Ma et al., 2024).

Beyond localized joint effects, the “gut-joint axis” has emerged as a focal point in the investigation of osteoarticular disorders. Gut dysbiosis and compromised intestinal barrier integrity trigger systemic low-grade inflammation, subsequently exacerbating localized articular degeneration (Sun et al., 2025). Recent investigations have demonstrated that these botanical metabolites possess the capacity to reshape the gut micro-ecosystem by augmenting short-chain fatty acid (SCFA) levels and fortifying the intestinal barrier (Zhang et al., 2025). This systemic metabolic regulatory circuit may effectively reduce the systemic pro-inflammatory cytokine load, complementing their localized joint protection.

Nevertheless, while our animal models highlight the promising disease-modifying osteoarthritis drug (DMOAD) potential of these botanical interventions, these findings should be interpreted with caution. Future translational studies are warranted to address the pharmacological barriers of low oral bioavailability and to conduct rigorous pharmacokinetic and safety evaluations in large animal models, thereby establishing a robust foundation for clinical trials.

### 4.1 Limitations

Several limitations of this meta-analysis warrant acknowledgment. First, the included animal studies exhibited substantial variations in modeling techniques, species selection, and intervention dosages; such heterogeneity may compromise the precision of the pooled effect size estimates. Furthermore, the primary literature pervasively lacks rigorous taxonomic authentication and explicit reporting of botanical sourcing, which introduces critical botanical ambiguity. Our methodological decision to extract data exclusively from the highest-dose cohorts introduced a maximization bias regarding the pharmacological effects. While this approach is instrumental in establishing the maximum proof-of-concept pharmacological effect in Murine subjects, it may overestimate the average pharmacological effects and obscure potential dose-response relationships or toxicity thresholds. In addition, the majority of the included studies lacked sufficient detail regarding rigorous methodological designs, particularly concerning allocation concealment and the blinding of outcome assessors. We must critically concede that this high risk of bias severely undermines the reliability of our findings. The absence of blinding, especially in subjective evaluations such as histological grading and behavioral pain metrics, significantly inflates the risk of measurement bias and carries a high probability of driving an overestimation of the true effect sizes. Finally, due to the limited number of independent studies available per specific outcome metric, calculating mathematically stable 95% prediction intervals was unfeasible, which may limit the precise forecasting of effect sizes for future translational research.

## 5 Conclusion

In conclusion, by synthesizing current preclinical evidence, this meta-analysis demonstrates that the genus *Epimedium* and its active botanical metabolites possess distinct multidimensional pharmacological potential in models involving Murine subjects with OA. These interventions exerted positive pharmacological effects in ameliorating cartilage surface morphology, evidenced by improved Pelletier scores, while concurrently suppressing core local inflammatory mediators such as IL-1β and IL-18, and alleviating thermal hyperalgesia. Although certain metrics, including the OARSI score, failed to achieve statistical significance under stringent statistical penalties, their highly consistent modulatory trends further corroborate the biological plausibility of utilizing this botanical drug for OA intervention. However, given the prevalent high risk of bias and substantial statistical heterogeneity inherent in current *in vivo* studies, the actual magnitude of the pharmacological effects when translating these preclinical proof-of-concept findings into clinical practice must be interpreted with caution. Looking ahead, large-scale, standardized *in vivo* experiments strictly adhering to the ARRIVE guidelines are urgently warranted to further substantiate their definitive translational value.

## Data Availability

The original contributions presented in the study are included in the article/Supplementary Material, further inquiries can be directed to the corresponding authors.

## Supplementary material

SUPPLEMENTARY APPENDIX 1 Search strategy.

SUPPLEMENTARY APPENDIX 2 GRADE summary of findings table.
