# Supplementary material for: Preclinical pharmacological effects of epimedium-derived interventions in osteoarthritis: a systematic review and meta-analysis
Source: Front Pharmacol. 2026 Aug 10;17:1828529. doi: 10.3389/fphar.2026.1828529 (PMC13500522; doi:10.3389/fphar.2026.1828529)
Supplement: Supplementary file 3 [file Supplementaryfile1.docx]

| **Database** | **Date of Search** | **Exact Search String / Query** | **Yield (n)** |
| --- | --- | --- | --- |
| **PubMed** | Oct 2025 | ((mouse OR mice OR murine OR rat OR rats OR rodent) AND ("Epimedium"[MeSH Terms] OR "Herba Epimedii" OR icariin) AND ("Osteoarthritis"[MeSH Terms] OR "Osteoarthritis" OR "OA" OR "degenerative joint disease")) | 23 |
| **Embase** | Oct 2025 | ('icariin'/exp OR 'icariin' OR 'epimedium'/exp OR 'epimedium' OR 'epimedii folium' OR 'yinyanghuo') AND ('rat'/exp OR 'rat' OR 'rats' OR 'mouse'/exp OR 'mouse' OR 'mice' OR 'murine') AND ('osteoarthritis'/exp OR 'osteoarthritis' OR 'oa' OR 'osteoarthritides') | 50 |
| **Web of Science** | Oct 2025 | TS=((mouse OR mice OR murine OR rat OR rats OR rodent) AND (epimedium OR "herba epimedii" OR icariin) AND (osteoarthritis OR "degenerative joint disease" OR OA)) | 62 |
| **FMRS** | Oct 2025 | (淫羊藿 OR 淫羊藿苷 OR 朝藿定 OR 宝藿苷 OR 双藿苷 OR 巫山淫羊藿) AND (鼠 OR 大鼠 OR 小鼠 OR 实验动物 OR 动物模型) AND (骨关节炎 OR 关节炎 OR 软骨退变 OR 软骨损伤 OR OA OR osteoarthritis) AND (软骨 OR 滑膜 OR 炎症因子 OR IL-1β OR TNF-α OR MMP-13 OR 焦亡 OR pyroptosis) NOT (细胞实验 OR 体外 OR 临床 OR 人 OR 病人 OR 患者) | 20 |
| **CNKI** | Oct 2025 | ( (SU='淫羊藿' OR TI='淫羊藿' OR KY='淫羊藿' OR AB='淫羊藿') OR (SU='Epimedium' OR TI='Epimedium' OR KY='Epimedium' OR AB='Epimedium') OR (SU='淫羊藿苷' OR TI='淫羊藿苷' OR AB='淫羊藿苷') OR (SU='icariin' OR TI='icariin' OR AB='icariin' OR AB='ICA') OR (AB='淫羊藿总黄酮' OR FT='淫羊藿总黄酮') OR (AB='宝藿苷' OR AB='baohuoside') ) AND (SU='骨关节炎' OR TI='骨关节炎' OR KY='骨关节炎' OR AB='骨关节炎' OR SU=' osteoarthritis' OR TI=' osteoarthritis' OR AB=' osteoarthritis' OR SU='OA' OR TI='OA' OR AB='OA') AND (SU='大鼠' OR TI='大鼠' OR KY='大鼠' OR AB='大鼠' OR SU='rat' OR TI='rat' OR AB='rat' OR SU='rats' OR TI='rats' OR AB='rats' OR AB='SD大鼠' OR AB='Wistar大鼠') | 32 |
| **WanFang** | Oct 2025 | (主题:淫羊藿 OR 题名:淫羊藿 OR 关键词:淫羊藿 OR 摘要:淫羊藿 OR 主题:仙灵脾 OR 题名:仙灵脾) AND (主题:骨关节炎 OR 题名:骨关节炎 OR 关键词:骨关节炎 OR 摘要:骨关节炎 OR 主题: osteoarthritis OR 题名:OA OR 关键词:关节退行性变) AND (主题:大鼠 OR 题名:大鼠 OR 关键词:大鼠 OR 摘要:大鼠 OR 主题:鼠 OR 题名:SD大鼠 OR 题名:Wistar大鼠) | 16 |
| **VIP** | Oct 2025 | (M=(淫羊藿) OR M=(仙灵脾) OR T=(淫羊藿) OR T=(仙灵脾) OR K=(淫羊藿) OR K=(仙灵脾) OR R=(淫羊藿) OR R=(仙灵脾) OR M=(icariin) OR T=(icariin) OR K=(icariin) OR R=(icariin)) AND (M=(骨关节炎) OR M=( osteoarthritis) OR M=(OA) OR T=(骨关节炎) OR T=( osteoarthritis) OR T=(OA) OR K=(骨关节炎) OR K=( osteoarthritis) OR K=(OA) OR R=(骨关节炎) OR R=( osteoarthritis) OR R=(OA)) AND (M=(大鼠) OR M=(鼠) OR M=(rat) OR M=(rats) OR T=(大鼠) OR T=(鼠) OR T=(rat) OR T=(rats) OR K=(大鼠) OR K=(鼠) OR K=(rat) OR K=(rats) OR R=(大鼠) OR R=(鼠) OR R=(rat) OR R=(rats) OR T=(SD大鼠) OR T=(Wistar大鼠)) | 15 |
