# Supplementary material for: Preclinical pharmacological effects of epimedium-derived interventions in osteoarthritis: a systematic review and meta-analysis
Source: Front Pharmacol. 2026 Aug 10;17:1828529. doi: 10.3389/fphar.2026.1828529 (PMC13500522; doi:10.3389/fphar.2026.1828529)
Supplement: Supplementary file 4 [file Supplementaryfile2.docx]

# GRADE Evidence Profile: Epimedium-Derived Interventions for Osteoarthritis (V2)

This document presents the standardized GRADE (Grading of Recommendations, Assessment, Development, and Evaluation) evidence profile table evaluating the certainty of preclinical evidence for epimedium-derived interventions in murine subjects modeled with osteoarthritis.

| **Outcome Category / Specific Outcome** | **No of Studies (Animals)** | **Risk of Bias** | **Inconsistency** | **Indirectness** | **Imprecision** | **Publication Bias** | **Effect Size [95% CI] & P-value** | **Certainty** |
| --- | --- | --- | --- | --- | --- | --- | --- | --- |
| **OARSI score** (Cartilage Histopathology) | 5 studies (N=90) | Serious^a^ | Serious^b^ | No serious | Serious^c^ | No serious^d^ | SMD = -7.15 [-15.31 to -1.01]  P = 0.07 | ⊕−−−  **Very Low** |
| **Pelletier score** (Cartilage Histopathology) | 2 studies (N=40) | Serious^a^ | No serious | No serious | No serious^e^ | No serious^d^ | SMD = -2.25 [-3.07 to -1.44]  P < 0.001 | ⊕⊕⊕−  **Moderate** |
| **Serum IL-6** (Systemic Inflammation) | 3 studies (N=50) | Serious^a^ | No serious | No serious | No serious^e^ | No serious^d^ | SMD = -9.24 [-18.10 to -0.37]  P = 0.04 | ⊕⊕⊕−  **Moderate** |
| **Serum TNF-α** (Systemic Inflammation) | 3 studies (N=54) | Serious^a^ | Serious^b^ | No serious | No serious | No serious^d^ | SMD = -5.04 [-12.35 to 2.26]  P = 0.18 | ⊕⊕−−  **Low** |
| **Serum IL-1β** (Systemic Inflammation) | 3 studies (N=54) | Serious^a^ | Serious^b^ | No serious | No serious | No serious^d^ | SMD = -11.40 [-31.57 to 8.78]  P = 0.27 | ⊕⊕−−  **Low** |
| **Serum MMP-13** (Catabolic Biomarker) | 3 studies (N=40) | Serious^a^ | Serious^b^ | No serious | No serious | No serious^d^ | SMD = -5.04 [-12.35 to 2.26]  P = 0.18 | ⊕⊕−−  **Low** |
| **Mechanical Pain (PWT)** (Nociceptive Behavior) | 2 studies (N=40) | Serious^a^ | No serious | No serious | Serious^c^ | No serious^d^ | SMD = 8.63 [-4.84 to 22.11]  P = 0.21 | ⊕⊕−−  **Low** |
| **Thermal Pain (PWL)** (Nociceptive Behavior) | 2 studies (N=40) | Serious^a^ | No serious | No serious | No serious^e^ | No serious^d^ | MD = 2.04 [0.60 to 3.49]  P = 0.01 | ⊕⊕⊕−  **Moderate** |
| **Intra-articular MMP-13** (Joint Protease) | 4 studies (N=70) | Serious^a^ | Serious^b^ | No serious | Serious^c^ | No serious^d^ | SMD = -6.91 [-13.78 to -0.04]  P = 0.15 | ⊕−−−  **Very Low** |
| **Intra-articular IL-18** (Local Inflammation) | 2 studies (N=30) | Serious^a^ | No serious | No serious | No serious^e^ | No serious^d^ | SMD = -14.33 [-18.18 to -10.47]  P < 0.001 | ⊕⊕⊕−  **Moderate** |
| **Intra-articular IL-1β** (Local Inflammation) | 2 studies (N=30) | Serious^a^ | No serious | No serious | No serious^e^ | No serious^d^ | SMD = -8.12 [-13.51 to -2.72]  P < 0.01 | ⊕⊕⊕−  **Moderate** |
| **Intra-articular MMP-1** (Joint Protease) | 3 studies (N=50) | Serious^a^ | No serious | No serious | No serious^e^ | No serious^d^ | SMD = -7.08 [-13.50 to -0.65]  P = 0.03 | ⊕⊕⊕−  **Moderate** |
| **Intra-articular NLRP3** (Inflammasome Pathway) | 2 studies (N=30) | Serious^a^ | No serious | No serious | Serious^c^ | No serious^d^ | SMD = -9.25 [-18.52 to 0.02]  P = 0.05 | ⊕⊕−−  **Low** |
| **Intra-articular Type II Collagen** (Matrix Component) | 3 studies (N=50) | Serious^a^ | Serious^b^ | No serious | No serious | No serious^d^ | SMD = 5.70 [-2.37 to 13.78]  P = 0.17 | ⊕⊕−−  **Low** |
| **Chondrocyte IL-1β mRNA** (Gene Expression) | 2 studies (N=30) | Serious^a^ | Serious^b^ | No serious | Serious^c^ | No serious^d^ | SMD = -16.82 [-39.66 to 6.02]  P = 0.15 | ⊕−−−  **Very Low** |
| **Synovial Fluid TNF-α** (Synovial Microenvironment) | 2 studies (N=40) | Serious^a^ | Serious^b^ | No serious | Serious^c^ | No serious^d^ | SMD = -2.04 [-4.60 to 0.53]  P = 0.12 | ⊕−−−  **Very Low** |
| **Synovial Fluid IL-1β** (Synovial Microenvironment) | 2 studies (N=40) | Serious^a^ | No serious | No serious | No serious^e^ | No serious^d^ | SMD = -2.11 [-3.52 to -0.71]  P < 0.01 | ⊕⊕⊕−  **Moderate** |

### Footnotes & Methodological Justifications

**a. Risk of Bias:** Downgraded by 1 level due to lack of explicit reporting on allocation concealment, random housing, and blinding of outcome assessors across the majority of included studies using the SYRCLE tool guidelines.

**b. Inconsistency:** Downgraded by 1 level for outcomes displaying excessive statistical heterogeneity (I² > 50% or P < 0.10). For outcomes where inconsistency is marked as "No serious", despite minor variance in effect magnitude, all individual study effect vectors remained highly symmetrical and point directions consistently favored the intervention arm, satisfying preclinical directionality consensus.

**c. Imprecision:** Downgraded by 1 level for outcomes that did not achieve traditional statistical significance due to the small preclinical sample sizes combined with conservative Knapp-Hartung standard error adjustments, causing the 95% CI to span across the line of no effect.

**d. Publication Bias:** Not downgraded. Although Egger linear regression indicated asymmetry driven by small-study effects, contour-enhanced funnel plots combined with robust trim-and-fill analysis (0 virtual studies required) confirmed the statistical resilience of the dataset against conventional selective non-reporting bias.

**e. Precision Preservation:** Not downgraded for imprecision because the effect estimation attained strong statistical significance (P < 0.05), possessed a large magnitude, and the confidence interval narrow bounds remained strictly detached from the null line.
